# Draft Genome Sequence of Sporolactobacillus inulinus NBRC 111894, Isolated from Kôso, a Japanese Sugar-Vegetable Fermented Beverage

**DOI:** 10.1128/MRA.00751-19

**Published:** 2019-10-10

**Authors:** Tai-Ying Chiou, Wataru Suda, Kenshiro Oshima, Masahira Hattori, Chiaki Matsuzaki, Kenji Yamamoto, Tomoya Takahashi

**Affiliations:** a Department of Biotechnology and Environmental Chemistry, Kitami Institute of Technology, Kitami, Hokkaido, Japan; b Laboratory for Microbiome Sciences, RIKEN Center for Integrative Medical Sciences, Yokohama, Kanagawa, Japan; c Department of Computational Biology, Graduate School of Frontier Sciences, The University of Tokyo, Kashiwa, Chiba, Japan; d Research Institute for Bioresources and Biotechnology, Ishikawa Prefectural University, Nonoichi, Ishikawa, Japan; e ARSOA Research & Development Center, AOB Keioh Group Corporation, Hokuto, Yamanashi, Japan; University of Maryland School of Medicine

## Abstract

Sporolactobacillus inulinus NBRC 111894 is a species of endospore-forming lactic acid bacteria isolated from kôso, a Japanese sugar-vegetable fermented beverage. The draft genome sequence of *S. inulinus* NBRC 111894 is useful for understanding the differences between S. inulinus strains and their conserved characteristics.

## ANNOUNCEMENT

Kôso is a Japanese fermented beverage made of various vegetables and a high concentration of sugar (1). Since its fermentation occurs spontaneously, originating in the natural microbial community in the raw materials, kôso contains a variety of microorganisms (1). In our previous studies, an unknown, predominant, and difficult-to-culture species was found in kôso (1). The new species was successfully isolated using a dilution-to-extinction technique. It was proposed as Lactobacillus kosoi sp. nov. (2), and its genomic information was reported (3). Other Lactobacillus species, such as Lactobacillus farciminis, were found and isolated from kôso, and their draft genome sequences were subsequently reported (4). Sporolactobacillus inulinus NBRC 111894 is a species of endospore-forming lactic acid bacteria (5) which was isolated from kôso after heat treatment (70°C for 1 h). For genomic DNA sequencing, pure culturing of *S. inulinus* NBRC 111894 was carried out in MRS broth (Difco Laboratories) under aerobic conditions (unmodified atmosphere) at 30°C for 2 days. The classical formula of MRS broth is according to de Man, Rogosa, and Sharpe (6). The genomic DNA was prepared as detailed in our previous report (1).

Whole-genome sequencing of *S. inulinus* NBRC 111894 was performed using an Ion Torrent PGM system. A total of 2,800,097 reads were assembled into 118 contigs using Newbler v2.8 (Roche). The largest contig size, average contig size, and contig *N*_50_ value were 174,007 bp, 26,056 bp, and 59,839 bp, respectively. The number of bases with a quality score of ≥Q40 was 3,073,441, which was 99.4% of the total contig length. The average read coverage for the genome was 272.75×. The average G+C content was 45.3%. Based on CheckM (7), the genome is estimated to be 94.83% complete. The draft genome of *S. inulinus* NBRC 111894, annotated by the RAST server (http://rast.theseed.org/FIG/rast.cgi) and using Glimmer3 with its default settings, contains 4,720 candidate open reading frames, 4 rRNA genes, and 60 tRNA genes.

The VISTA servers (http://genome.lbl.gov/cgi-bin/WGVistaInput) (8, 9), with their default settings, were used for gene comparisons, with the Sporolactobacillus inulinus CASD (10) genome being applied as a reference. *S. inulinus* NBRC 111894 showed three annotated genes relating to lipopolysaccharide biosynthesis and metabolism; in contrast, *S. inulinus* CASD showed only one. Genes relating to the CRISPR/Cas system (11) were found in both *S. inulinus* NBRC 111894 (*csn1* and *cas1*) and *S. inulinus* CASD (*cas1*, *cas2*, *cas3*, *cas4*, *cas5*, and *cas6*). The genes associated with antibiotic resistance, annotated as those for multidrug resistance protein B, spectinomycin phosphotransferase, streptomycin 6-kinase, and teicoplanin resistance protein, were found in *S. inulinus* NBRC 111894 but not in *S. inulinus* CASD. The two strains therefore showed different means of achieving environmental adaptability. The genomic information thus appeared to be helpful in the further understanding of *S. inulinus.*

### Data availability.

The genome sequence of Sporolactobacillus inulinus NBRC 111894 has been deposited in DDBJ/EMBL/GenBank under accession number BEXB00000000. The version described in this paper is version BEXB01000000. The Sequence Read Archive (SRA) of Sporolactobacillus inulinus NBRC 111894 has been deposited via the DDBJ system, and the accession number has been released as DRR153268.
